# Media Supplementation With Gamma‐Oryzanol Improves the Outcome of Ovine Oocyte Maturation In Vitro

**DOI:** 10.1002/vms3.70134

**Published:** 2024-12-17

**Authors:** Saeed Musapoor, Najmeh Davoodian, Ali Kadivar, Ebrahim Ahmadi, Hassan Nazari

**Affiliations:** ^1^ Research Institute of Animal Embryo Technology Shahrekord University Shahrekord Iran; ^2^ Department of Clinical Sciences Faculty of Veterinary Medicine Shahrekord University Shahrekord Iran

**Keywords:** antioxidant, gamma‐oryzanol, mitochondria, ovine, transcription

## Abstract

**Background:**

The process of maturing ovine oocyte in vitro has not yet been raised with acceptable results.

**Objective:**

This study was designed to evaluate the γ‐oryzanol effect as a supplement of maturation media on the development of ovine oocytes to blastocyst.

**Methods:**

Aspirated from ovine ovaries, morphologically normal cumulus‐oocyte complexes (COCs) were matured in media supplemented with or without 5 µM γ‐oryzanol. Matured oocytes were divided into two parts: one evaluated for their nuclear maturation, the level of GSH and ROS, mitochondrial membrane potential (MMP) and the pattern of transcription in oocytes and respective cumulus cells (CCs), and another subjected to fertilisation and culture to assess the development of oocytes to the blastocyst.

**Results:**

γ‐Oryzanol improved the proportion of cleaved embryos and total blastocysts in the treated group, which was linked to improved MMP, higher levels of intracellular GSH and lower levels of ROS. A lower proportion of MI and GVBD was recorded for treated oocytes in comparison with control, although the proportion of MII oocytes was not different between groups. The treated oocytes and CCs showed downregulation of genes related to apoptosis (*BAX* and *CASP‐9*) and upregulation of genes related to antioxidative status (*NRF2*, *CAT* and *SOD*). In conclusion, our results demonstrated the improved developmental outcome of supplemented oocytes so that the antioxidant response and higher enzymatic activity were maintained, and the generation of ROS was turned off; therefore, a novel alternative for counteracting oxidative stress in ovine oocytes undergoing maturation was offered by γ‐oryzanol through an antioxidative pathway.

## Graphical abstract

This study demonstrated the γ‐oryzanol effect as a supplement of maturation media on the development of ovine oocytes to blastocyst through an antioxidative pathway to sustain the antioxidant cellular response, higher enzymatic activity, turn off ROS generation and provide a valid first line of defence against oxidative stress.

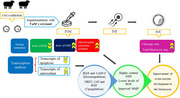

## 1 Introduction

Small ruminants provide an important food source; however, the efficiency of traditional breeding systems is not high enough to meet the increasing demand for meat and milk (Sargison 2020). It is well known and accepted that advanced reproductive techniques improve reproduction efficiency and genetic acceleration (Souza‐Fabjan et al. 2023), and the most applied technique worldwide for production improvement in livestock is the process of in vitro embryo production (IVEP), which is still the leading technique in small ruminants (Souza‐Fabjan et al. 2023). This technique is an extremely vital basic tool for establishing other biotechnologies, including cloning and transgenesis (Souza‐Fabjan et al. 2021). However, this trend is not as advanced as in cattle due to lower commercial value, and fewer professional and research interest in this area (Souza‐Fabjan et al. 2021). IVEP is not very efficient yet in small ruminants, although many attempts have been made to improve the outcome. This process involves several consequent steps of oocyte and embryo manipulation in vitro in which the cells are exposed to exogenous and endogenous factors responsible for the free radical formation (Cognie et al. 2003). The overproduction of reactive oxygen species (ROS) unable to be scavenged in cells results in oxidative stress, which is responsible for damage to macromolecules, membranes and organelles in oocytes and embryos, impairs their quality and potential to develop and causes suboptimal outcomes (Agarwal, Durairajanayagam, and Du Plessis 2014).

To date, many efforts have been made to improve the efficiency of ovine IVEP, focusing on counteracting oxidative stress. Meanwhile, as a nice and interesting area of work, supplementing the media with antioxidants for their scavenging potential has received more attention. In this context, numerous antioxidants have been evaluated during the development of ovine oocytes and embryos, and controversial results have been reported (Wang et al. 2002; Zabihi et al. 2019). The effect of antioxidant supplementation of maturation media on the efficiency of ovine oocyte development has been extensively evaluated. Supplementation of maturation media with CoQ10, alpha‐tocopherol (200 µM), sodium selenite (40 µM), melatonin (30 µM) and ascorbic acid (100 µM) has been demonstrated to improve the capacity of ovine oocyte development and embryo quality (Heydarnejad et al. 2019; Tripathi et al. 2023). Moreover, the resveratrol supplement was shown to reverse the cadmium‐induced oxidative stress in maturing ovine oocyte through reduction of ROS, improvement of mitochondrial function, cytoskeleton morphology and cortical granule distribution, and also promoting the transcription of molecular targets including SIRT1, SOD1 and GPX1, all leading to the enhancement of oocyte maturation and fertilisation (Piras et al. 2022).

Recently, γ‐oryzanol, a component extracted from rice bran oil, has been noticed as an anti‐inflammatory, anti‐diabetic, anti‐tumour and antioxidant agent (Akihisa et al. 2000; Juliano et al. 2005). It has been introduced as a free radical scavenger and an endogenous antioxidant enzyme activity modulator (Moon et al. 2017). Moreover, the antioxidative and antiapoptotic potential of γ‐oryzanol has been well studied in vivo and in vitro (Chanapiwat and Kaeoket 2015; Huang et al. 2020; Rungratanawanich et al. 2018). In a previous study, the effect of γ‐oryzanol on the development of ovine oocytes and embryos was evaluated and gave rise to outstanding results (Musapoor et al. 2023), which showed a significant positive effect of the γ‐oryzanol supplement on in vitro ovine oocyte and embryo development, and introduced the optimal concentration of γ‐oryzanol in maturation and culture media. However, there is no study yet regarding the mechanism underlying the effect of γ‐oryzanol on ovine oocytes that mature in vitro. So based on the significant role of γ‐oryzanol in improving IVM outcomes, we hypothesised that supplementing the maturation media with γ‐oryzanol might benefit the antioxidant defence mechanism of ovine oocytes. Therefore, the present study aimed to evaluate the effect of γ‐oryzanol on oocyte nuclear maturation and subsequent development, mitochondrial membrane potential, the levels of ROS and GSH, and quantitative changes of transcription in matured oocytes and their respective CCs during IVM, as a basic and important stage of embryo production in vitro.

## 2 Materials and Methods

The powder of γ‐oryzanol (Sigma‐Aldrich; CDS021604) was dissolved in ethanol, which remained less than 1% in culture media. All the other chemicals in this study were obtained from Sigma Chemicals Co. (St. 85 Louis, MO, USA) except for those stated.

### 2.1 Recovery and Maturation of Oocytes

The study was carried out between November and March 2023. The ovaries of slaughtered ewes were kept in a warmed saline solution and transported to the laboratory, where morphologically normal medium‐sized follicles (2–6 diameter) (Lorenzo‐Torres et al. 2023; Wani et al. 2013) were subjected to aspiration using a vacuum pump (JP‐40 V KAWAKE, Taiwan). Aspiration media were composed of HEPES‐buffered media 199 (HTCM) supplemented with 5% FBS and 100 IU/mL heparin. COCs were evaluated microscopically (Olympus CKX41, Japan) to select morphologically normal ones. Evenly granulation of ooplasm, the presence of at least three layers of cumulus cells and no signs of degeneration and expansion were considered as the criteria for selection. Selected COCs were washed three times in FBS‐supplemented HTCM, and then transferred into droplets of preincubated maturation media composed of bicarbonate‐buffered medium 199, 0.33 mM sodium pyruvate, 0.05 IU/mL FSH and 12% FBS. The COCs were randomly subjected to culture in media supplemented with 5 µM γ‐oryzanol as treatment (ORY‐5), or without treatment as Ctrl, 50 µL droplets 10 each, under mineral oil in a humidified atmosphere (Labotect C60, Germany), 5% CO_2_ for 22 h at 38.5°C. Four biological replications were included. The concentration of γ‐oryzanol in maturation media was determined based on a previous study (Musapoor et al. 2023).

### 2.2 In Vitro Fertilization and Culture

At the end of IVM, matured COCs were evaluated for the expansion of the cumulus cover; a subjective scoring system was used for describing the expanded cumulus, where all layers of cumulus cells including those closest to the oocyte expanded (Boni, Cuomo, and Tosti 2002).

A modified Tyrode preparation containing 25 mM sodium bicarbonate, 0.6% bovine serum albumin, 10 mM lactate, 1.0 mM pyruvate and 5.6 mM glucose as IVF‐TALP was prepared for culture of matured COCs to be fertilised. The ram testes were collected from the Slaughterhouse, and fresh sperm was collected from the tail of the epididymis, suspended in 0.4% BSA‐supplemented HTCM for 1 h, and next in 0.4% BSA‐supplemented HEPES‐SOF for 20 min at 39°C. Morphologic evaluation was performed to ensure sperm motility, and swim‐up separation of highly motile spermatozoa was performed through centrifugation (Eppendorf 5424, Germany) of the upper fraction at 200 × g for 2 min, which was coincubated with COCs (5000 sperm per oocyte), under mineral oil at 39°C and 5% CO_2_ in a humidified atmosphere.

On the day after IVF (Day 1; Day 0 = the day of fertilisation), presumptive zygotes were denuded from the cumulus cover and evaluated microscopically to determine the degenerated oocytes to be discarded. All the surviving presumptive zygotes were transferred to 20 µL droplets of synthetic oviductal fluid (SOF) media supplemented with amino acids and 0.8% BSA, six zygotes each, incubated at 39°C, in a humidified atmosphere with 5% CO_2_ and 7% O_2_. Two days later (third day), the cleaved embryos were recorded and transferred to droplets of fresh culture media supplemented with 10% charcoal‐stripped FBS and kept until the seventh day to evaluate and record the number of blastocysts. Four biological replications were included.

### 2.3 Assessment of Nuclear Maturation

At the end of IVM, oocytes were denuded from cumulus cover by gentle manipulation to be stained with Hoechst 33342 for 5 min. Evaluation for nuclear maturation status was made using an epifluorescent microscope (Olympus, Japan). The observation of a polar body (PB) in perivitelline space (PVS) or two shiny spots in ooplasm were recorded as metaphase II (MII) stages, while irregular networks of condensed chromatin or metaphase plate were recorded as metaphase I (MI). Clumped or slightly condensed chromatin was considered as germinal vesicle breakdown (GVBD), and those with fluorescent foci different from other groups were considered as fragmented.

### 2.4 Levels of Reactive Oxygen Species and Glutathione (GSH)

The evaluation of ROS and GSH was performed according to the previous studies (Heydarnejad et al. 2019). At the end of maturation, the denuded oocytes from each group (*n *= 100 per group from three replications) were exposed to 10 µM 2,7‐dichloro dihydroflourescein diacetate and 10 µM Cell Tracker Blue CMF2HC (Cell Tracker Blue; Invitrogen Corporation, Carlsbad, CA, USA) and incubated at 38.5°C in the dark for 20 min, then washed and placed into 10 µL drops of PBS/PVA, finally observed under an epifluorescence microscope and imaged immediately. B‐2E/C (ROS) and UV‐2A (GSH) filters were employed to capture the emitted fluorescence, and then the relative fluorescence intensity of images was analysed by Image software (National Institutes of Health, Bethesda, MD), as described previously (Igarashi et al. 2016). Briefly, the level of fluorescent intensity was determined through Image J by circling each individual oocyte in images using a drawing tool to limit the area of interest. Calculating the corrected total cell fluorescence (CTCF) was done using the formula (CTCF = integrated density − [area of selected cell × mean fluorescence of background readings]).

### 2.5 Mitochondrial Membrane Potential Measurement

For measurement of MMP, a lipophilic cationic dye, JC‐1 (Cat. No. M34152; Invitrogen, Carlsbad, CA), was used, which forms J‐aggregates and reflects red fluorescent when the membrane potential is high, while staying monomers and reflecting green when the potential is low. The red‐to‐green fluorescence ratio was measured in each oocyte individually for assessment of MMP. Matured denuded oocytes (*n* = 60 oocytes per group in three replicates) were exposed to JC‐1 probe (20 µg/mL) and incubated at 38.5°C for 30 min. Next, they were washed in PBS/PVA, placed individually in droplets of PBS/PVA, and observed under inverted fluorescence microscopy. Digital images were analysed by Image J software to accomplish relative quantification of fluorescence intensity.

### 2.6 Transcription Analysis

For this purpose, 25 COCs at the end of maturation were subjected to gentle manipulation to denude oocytes from the cumulus cover. Then pooled oocytes and respective CCs were stored in liquid nitrogen separately (four replications). The extraction of total RNA was performed using FavorPrepTM Blood/Cultured Cell Total RNA (Favorgen, Ping‐Tung, Taiwan). The user manual was followed step by step. In the first stage, the lysis was performed using 350 µL of FRAB buffer and 3.5 µL of β‐mercaptoethanol reagent. This mixture was loaded in a filter column and was centrifuged at full speed for 2 min to separate the supernatant, which was transferred to a new collection tube. A volume of ethanol (70% v/v) was added and mixed with the supernatant and filtered again. It was followed by three consequent washings of the filter with three different washing solutions. The next step, drying, was performed through centrifugation. Finally, the extracted RNA was eluted into RNase‐free distilled water, which was subjected to cDNA synthesis immediately. A spectrophotometer (LSPR spectrophotometer MPANM96, Nanomabna, Iran) was used for quantification and qualification, where the ratio of A260/280 ≥ 1.8 for extracted RNA was determined to proceed to the next step.

Reverse transcription was performed using M‐MLV reverse transcriptase (YektaTajhizAzma, Iran). A mixture composed of 1 µL of extracted RNA, 0.5 µL random hexamer and 0.5 µL oligo (dT), in total 13.5 µL, was prepared and incubated at 70°C for 5 min. After chilling on ice, another mixture composed of 4 µL 5× first strand buffer, 1 µL dNTPs, 0.5 µL RNsin and 1 µL M‐MLV, in total 6.5 µL, was added. The final mixture in total volume of 20 µL was incubated at 37°C for 60 min and finally was terminated by 5 min heating at 70°C.

Rotor‐Gene Q 6000 (Qiagen, Hilden, Germany) was used for quantitative real‐time PCR, which was performed in triplicate manner for each sample. A mixture was prepared composed of 10 µL of SYBR master mix (RelQ Plus 2x Master Mix Green; Ampliqon, Denmark), 1 µL synthesised cDNA and 0.5 µM of each specific primer, which is listed in Table 1. DNase‐free distilled water was added to the mixture to reach the total volume of 20 µL.

**TABLE 1:** The information on primers used for RT‐PCR.

Gene primer	Primer sequences	Melting point (°C)	Product size (bp)	Reference or accession gene bank
*SOD1*	F:TCATGGGTTCCACGTCCAT	51.1	62	Yang et al. (2013)
	R:GAGGGCCTGCACTGGTACAG	57.9		
*Catalase*	F:GCTCCAAATTACTACCCCAATAGC	55.7	104	Côrtes et al. (2012)
	R:GCACTGTTGAAGCGCTGTACA	54.4		
*Caspase9*	F:GCCAAGCCAAGGAAAACTCG	53.8	236	Han et al. (2020)
	R:CACGGCAGAAGTTCACGTTG	53.8		
*BAX*	F:GTTGTCGCCCTTTTCTACTTTGC	55.3	89	Sánchez‐Ajofrín et al. (2022)
	R:CAGCCCATGATGGTCCTGATC	56.3		
*BCL2*	F:GCGGCCCCTGTTTGATTTC	53.2	99	XM_018039337.1
	R:TTATGGCCCAGATAGGCACCC	56.3		
*NRF2*	F:TGTGGAGGAGTTCAACGAGC	53.8	88	Zhao et al. (2017)
	R:CGCCGCCATCTTGTTCTTG	53.2		
*Actin*	F:CTCTTCCAGCCTTCCTTCCT	53.8	178	BC102948
	R:GGGCAGTGATCTCTTTCTGC	53.8		
*GAPDH*	F:ATGGGCGTGAACCACGAGAA	53.8	146	NM 001190390
	R:ATGGCGTGGACAGTGGTCAT	53.8		

The reaction was held at 95°C for 15 min. Then thermocycling was set at 15 s at 95°C, 30 s at 60°C and 30 s at 72°C. After 40 cycles, the reaction was stopped, and the analysis of the melting curve was performed to determine the homogeneity of the products; the temperature ramp of 70°C–95°C was considered.

The normalisation of transcription data was performed based on *GAPDH* and *B‐Actin* as internal reference genes. The efficiency of the reaction was determined by LinRegPCR software version 2012.0 (Amsterdam, Netherlands). The mean efficiency value of individual genes was calculated using their amplification profiles, then the expression ratio was calculated using REST software (http://rest.gene‐quantification.info), which determined the statistical analysis to explore the differences between groups.

### 2.7 Statistics

All the proportional data were subjected to an arcsine transformation. SPSS statistical software package, version 20.0 (SPSS Inc., Chicago, IL, USA) was employed for analysis. The tests to highlight the differences between treatments consisted of a one‐way ANOVA followed by the post hoc Tukey test. The data of oocyte staining were analysed using an independent‐sample *t*‐test. The differences at the level of *p* < 0.05 were considered significant. The method for analysis of data related to relative transcription is mentioned above.

## 3 Results

### 3.1 The Effect of γ‐Oryzanol on Ovine Oocyte Nuclear Maturation

The results are presented in Table 2. The proportion of MI and GVBD oocytes was significantly lower in treatment compared to Ctrl (*p* < 0.05). However, the proportion of GV and fragmented oocytes as well as oocytes that reached MII stage was not different between groups.

**TABLE 2:** The effect of γ‐oryzanol on the post‐IVM nuclear maturation of ovine oocytes.

Treatments	No. of oocytes	GV (% ± SEM)	GVBD and MI (% ± SEM)	MII (% ± SEM)	Frag (% ± SEM)
ORY‐5	121	6 ± 0.02^a^	3 ± 0.02^a^	88 ± 0.03^a^	2 ± 0.0^a^
Ctrl	120	6 ± 0.02^a^	11 ± 0.03^b^	79 ± 0.04^a^	4 ± 0.02^a^

*Note*: Ovine COCs matured in media supplemented with 5 µM γ‐oryzanol (ORY‐5) and no supplementation (Ctrl). Mean percentages with different superscripts within each column differed (*p* < 0.05).

Abbreviations: Frag, fragmented oocytes; GV, germinal vesicle; GVBD, germinal vesicle breakdown; MI, metaphase I; MII, metaphase II.

### 3.2 The Effect of γ‐Oryzanol on Ovine Oocytes Development to Embryo

The results are presented in Table 3. The cumulus expansion of COCs in two groups seemed not to differ significantly. No significant effect on the proportion of survived oocytes at the end of fertilisation was detected.

**TABLE 3:** The effect of γ‐oryzanol on the development of ovine oocytes.

Treatments	No. of COCs	Expanded COCs (% ± SEM)	Survived zygotes (% ± SEM)	Cleaved embryos (% ± SEM)	Total blastocysts (% ± SEM)
ORY‐5	324	94 ± 0.02^a^	91 ± 0.02^a^	69 ± 0.04^a^	47 ± 0.04^a^
Ctrl	302	95 ± 0.02^a^	92 ± 0.02^a^	55 ± 0.03^b^	26 ± 0.03^b^

*Note*: ORY‐5 (matured in media supplemented with 5 µM γ‐oryzanol) and Ctrl (no supplementation during maturation); expansion, survival, cleavage, and total blastocyst rates were evaluated on Days 0, 1, 3, and 7, respectively. Day 0 = the day of IVF. Mean percentages with different superscripts within each column differed (*p* < 0.05).

The proportions of zygotes that cleaved and total blastocysts were significantly higher in treatment than in the Ctrl group (*p* < 0.05).

### 3.3 The Effect of γ‐Oryzanol on ROS and GSH Levels

**FIGURE 1 (:**
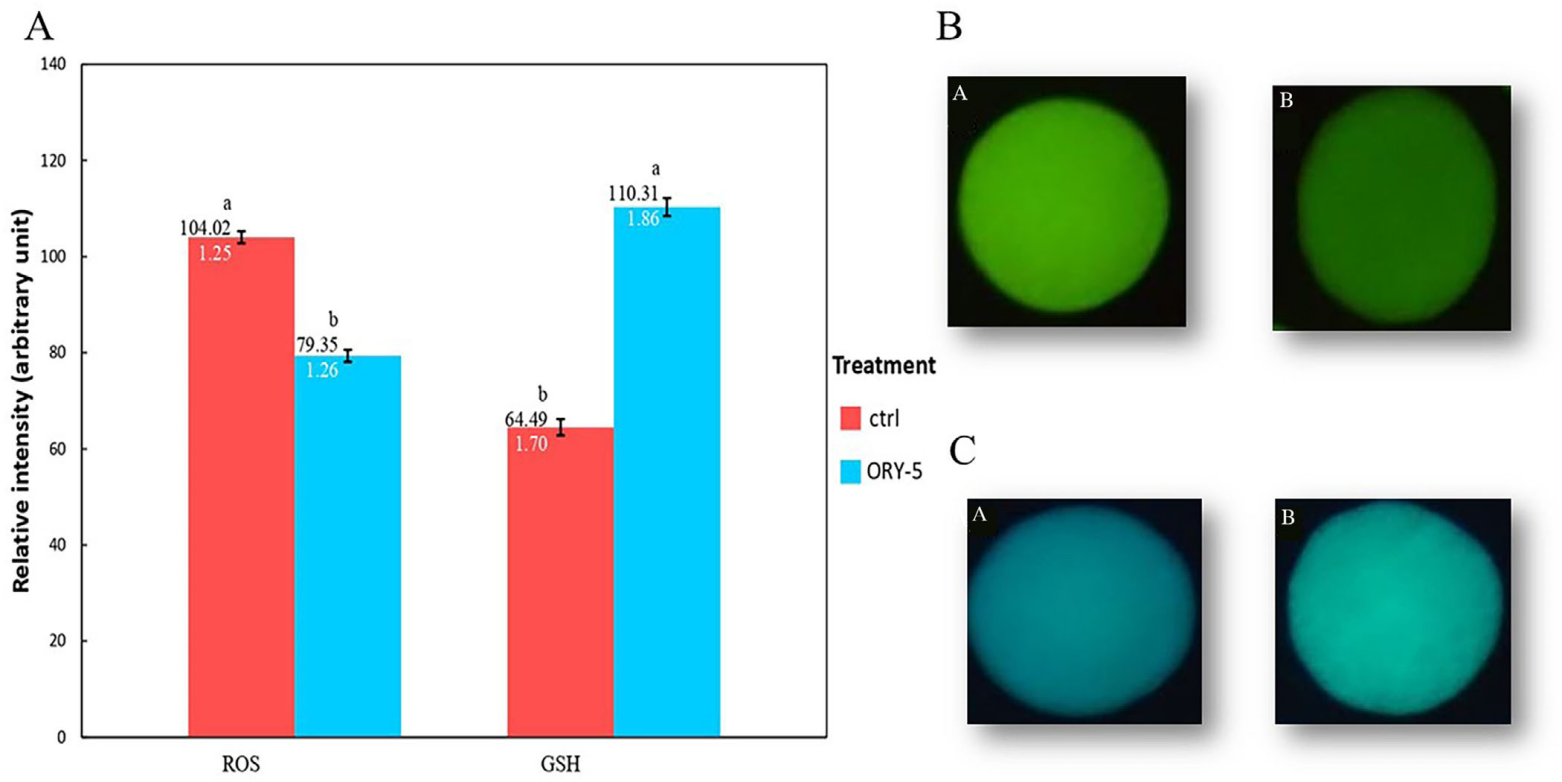
A) Relative intensity (expressed as arbitrary fluorescence units) of ROS and GSH levels in matured ovine oocytes in the presence or absence of γ‐oryzanol. (B) Representative fluorescence images of the ovine oocyte from control (a) and treatment (b) groups for ROS levels (green). (C) Representative fluorescence images of the ovine oocyte from control (a) and treatment (b) groups for GSH contents (blue).

An increase in the mean relative intensity of GSH was observed in treatment compared to Ctrl (110.31 ± 1.8 vs. 64.49 ± 1.7; *p* ˂ 0.05; Figure 1). A decrease in the mean relative level of ROS was detected in treatment compared to Ctrl (79.35 ± 1.26 vs. 104.02 ± 1.25; *p* ˂ 0.05; Figure 1).

### 3.4 The Effect of γ‐Oryzanol on Mitochondrial Inner Membrane Potential

**FIGURE 2:**
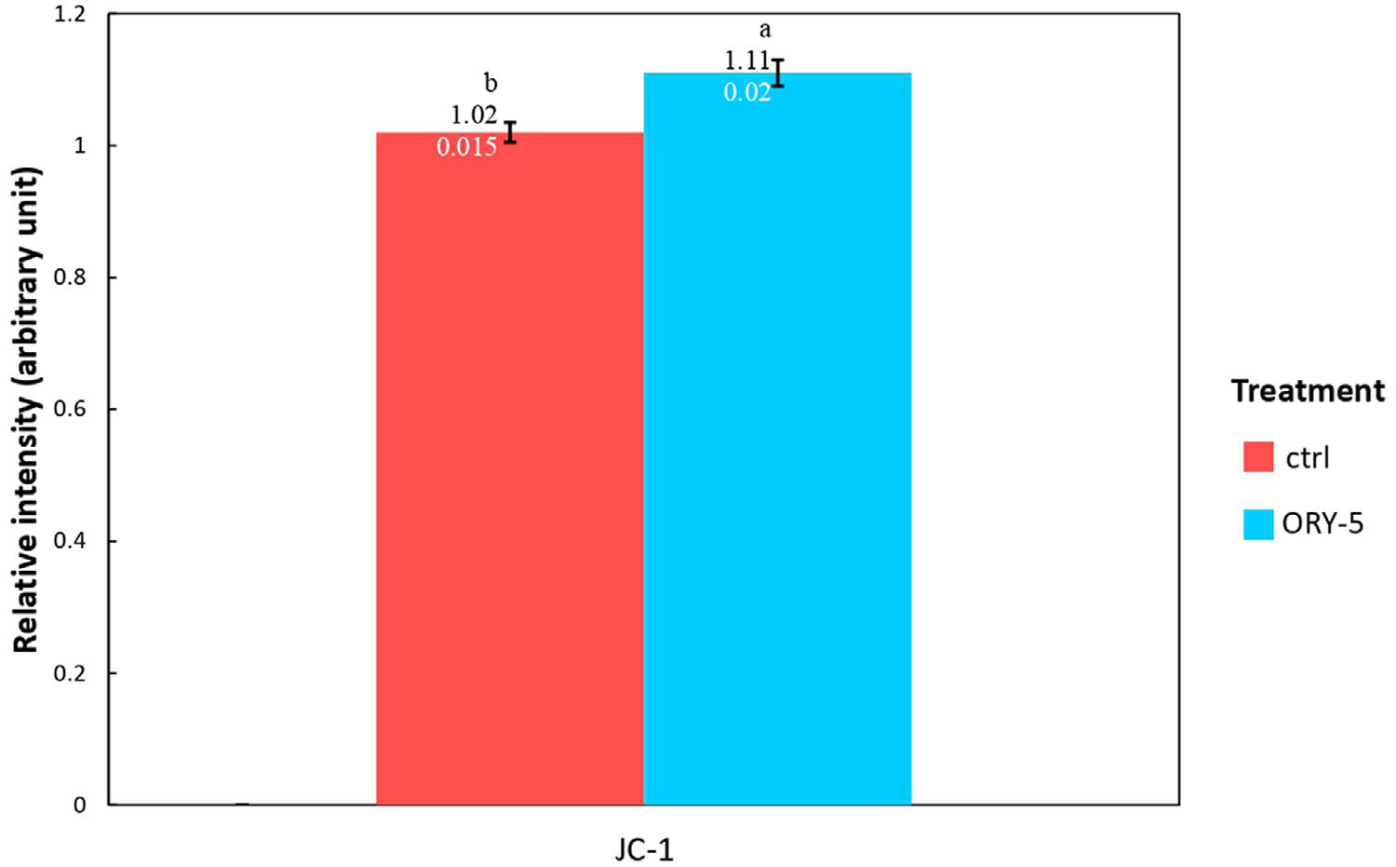
Relative ratio of JC‐1 fluorescence, in ovine oocytes matured in the presence or absence of γ‐oryzanol.

Results, present in Figure 2, revealed the significantly higher mitochondrial membrane potential of treated oocytes in comparison with the control group (1.11 ± 0.19 vs. 1.02 ± 0.14; *p* ˂ 0.05).

### 3.5 The Effect of γ‐Oryzanol on Relative Gene Expression

**FIGURE 3:**
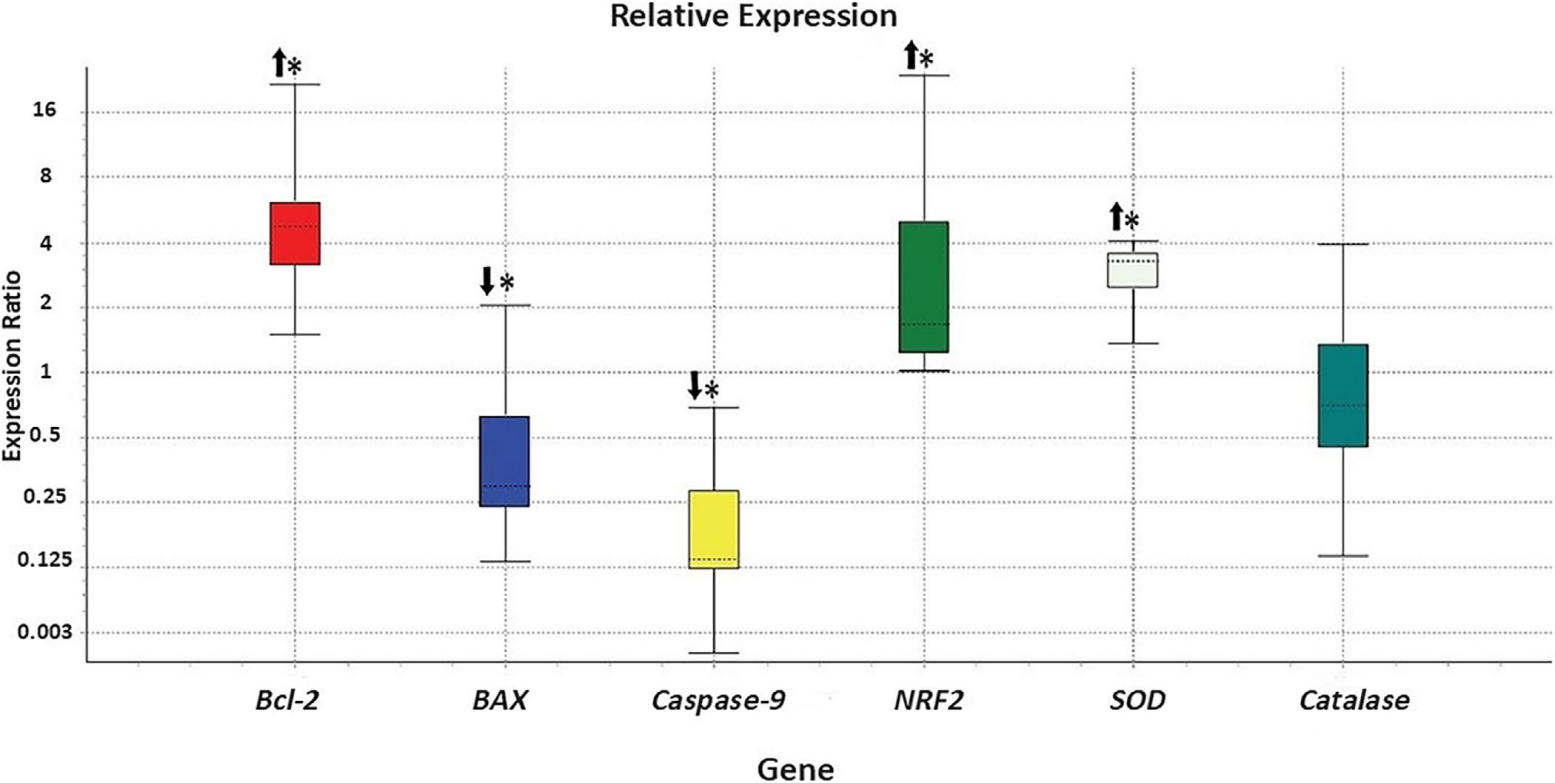
Relative abundance of transcripts for candidate genes in the oocytes of groups treatment and ctrl. The significant difference in the relative expression of genes is indicated by an asterisk (*). The boxes demonstrate the interquartile range. The dotted line indicates the median gene expression. The whiskers represent the minimum and maximum observations. (Results from four replicates.)

**FIGURE 4:**
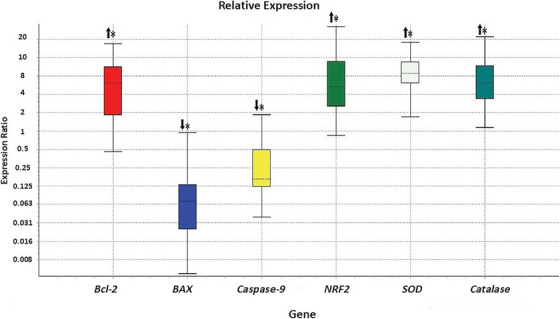
Relative abundance of transcripts for candidate genes in the CCs of groups treatment and ctrl. The significant difference in the relative expression of genes is indicated by an asterisk (*). The boxes demonstrate the interquartile range. The dotted line indicates the median gene expression. The whiskers represent the minimum and maximum observations. (Results from four replicates.)

To evaluate the apoptosis, *BCL‐2*, *BAX* and *CASP‐9* were selected. Comparing with Ctrl, treatment with *γ‐oryzanol* induced upregulation of *BCL‐2 and* downregulation of *BAX* and *CASP‐9* in oocytes (Figure 3) and CCs (Figure 4).

To evaluate the enzymatic antioxidant defence, *NRF2*, *SOD* and *CAT* genes were selected. In comparison with control, *γ‐oryzanol* treatment made upregulation of all selected transcripts in oocytes (Figure 3), and *NRF2* and *SOD* in CCs (Figure 4).

## 4 Discussion

Upon our results, the supplementation of maturation media with *γ‐oryzanol* made an improvement in the developmental competence of oocytes and enhanced the proportion of cleaved embryos and total blastocysts. The process of maturation involves a series of nuclear and cytoplasmic maturations that make the oocytes acquire the capacity for fertilisation and subsequent development. Meanwhile, oocytes are exposed to exogenous and endogenous factors that suppress the antioxidant defence mechanism, which results in oxidative stress (Agarwal et al. 2006). It is well demonstrated that during IVM, the competence of the maturing oocyte is compromised by the high levels of ROS, since ROS induces the apoptotic cascade, fragmentation of DNA, errors in spindle formation, impairs nuclear maturation, mitochondrial function and ATP production (Mihalas et al. 2017). Moreover, OS‐induced senescence in oocytes alongside lipid peroxidation‐induced damage to the sperm membrane compromises the fusion process of sperm and oocyte (Aitken 2020). On the other hand, ROS decreases the viability of CCs and prevents them from protecting oocytes and carrying out their role in the enrichment of oocytes (Russell et al. 2016). ROS is usually produced in living organisms; nevertheless, cells have antioxidant enzyme systems to scavenge free radicals. The imbalance of efficiency of this system and the overproduction of free radicals result in oxidative stress (Fang, Yang, and Wu 2002).

As an antioxidant, *γ‐oryzanol* has been demonstrated in numerous in vivo and in vitro experiments to induce the activity of endogenous antioxidative enzymes to scavenge free radicals and prevent oxidative stress (Islam et al. 2011; Minatel et al. 2016).

The mechanism underlying the antioxidative activity of γ‐oryzanol has been well investigated; *γ‐oryzanol* modulates the nuclear factor erythroid‐related factor 2 (NRF2) pathway, as the main regulator of antioxidant defence mechanism in cells, to induce the activity of endogenous enzymes for scavenging free radicals and prevent the oxidative stress (Amin et al. 2014). It is well known that under in vitro conditions, OS, which defines the condition of an imbalance between intracellular concentration of ROS and the capability of the antioxidant mechanism for ROS scavenging, negatively affects the maturation of oocytes and the development of embryos (Guerin, El Mouatassim, and Menezo 2001); the gene expression pattern (Balasubramanian et al. 2007) and the quality of embryos (Sudano et al. 2011). Therefore, oocytes and embryos need to activate some protective mechanisms (Takahashi 2012); amongst the NRF2‐mediated oxidative‐stress‐response pathway introduces the dominant responsive cascade (Gad et al. 2012), which regulates many downstream antioxidant enzymes in cells (Nguyen, Nioi, and Pickett 2009; Tanaka et al. 2008), including glutathione peroxidase (GPX), catalase (CAT) and superoxide dismutase (SOD), which have been shown to protect embryos from OS by scavenging ROS (Abedelahi et al. 2010; Ozawa et al. 2006); the upregulation of named genes alongside lower levels of ROS and higher GSH and MMP was observed in our experiments.

On the other hand, *γ‐oryzanol* might help the stabilisation of cell membranes, since a constituent of *γ‐oryzanol*, phytosterol plays the role of cholesterol, which forms an important part of the cell membrane structure and is essential for the synthesis of biomolecules such as steroids in living organisms (Kritchevsky and Bonfield 2012). Moreover, it has been hypothesised that hydroxy and phenoxy groups of γ‐oryzanol bind to free electrons of ROS and decrease their effects; it might be a mechanism for reducing the ROS‐induced lipid peroxidation of cell membranes (Cicero and Gaddi 2001). In line with these properties of γ‐oryzanol, our results demonstrated the improvement in the outcome of IVM and IVP, which demanded a detailed analysis relevant to the underlying mechanisms to be considered.

Upon our results, the expression of *NRF2*, *SOD* and *BCL‐2* was upregulated, and *BAX* and *CASP‐9* downregulated in treated oocytes; that confirms a lower level of apoptosis in the presence of γ‐oryzanol, and was in line with higher and lower intracellular levels of GSH and ROS, respectively. Poor developmental competence of in vitro matured oocytes may be caused by improper cytoplasmic maturation, which depends on the maturation environmental condition (Marchal et al. 2001). The intracellular level of GSH has been introduced as a criterion for the prediction of cytoplasmic maturation of oocytes (Luberda 2005). A low level of GSH in oocytes has been linked to compromised development; the degree of cytoplasmic maturation determines the competence of monospermic fertilisation and developmental potential of early embryos up to embryonic genome activation (EGA) (Koo et al. 2005). GSH in mature oocytes has a critical role in the decondensation of sperm chromatin to form the male pronucleus (Luberda 2005). Consistent with the above, we observed greater levels of GSH in treated oocytes, which also improved developmental outcomes. These results suggest *γ‐oryzanol* as a useful IVM supplement that improves cytoplasmic maturation and subsequent fertilisation and development. Alongside, the level of intracellular ROS was low in treated oocytes; this might be related to the direct effect of the antioxidant reducing ROS production leading to lower consumption of GSH to scavenge excessive ROS.

On the other hand, non‐enzymatic pathway through GSH, *γ‐oryzanol* accomplishes its antioxidative properties through an enzymatic mechanism too. Interestingly, this has been approved by the upregulation of *NRF2* and *SOD* transcripts in treated oocytes with low and high levels of ROS and GSH. It was in line with the results of a previous study in somatic cells that introduced *γ‐oryzanol* as an effective scavenger of ROS in cells (Huang et al. 2020). These findings suggest that supplementation of IVM media with *γ‐oryzanol* provides a suitable in vitro environment for the maturation and subsequent development of ovine oocytes. On the other hand, MMP was improved in treated oocytes in our study which is very interesting since mitochondria have an important role in energy production and contribute to proper chromosomal segregation, which is vital during the final stage of meiosis when the oocyte is activated by sperm entry (Eichenlaub‐Ritter et al. 2004). In line with this, γ‐oryzanol has been demonstrated to block the ROS‐activated mitochondrial apoptotic pathway, inhibit cell apoptosis and alleviate cytotoxicity in hepatic cells (Huang et al. 2020).

Our results demonstrated the upregulation of *BCL‐2, NRF2, SOD* and *CAT*, and the downregulation of *BAX* and *CASP‐9* transcripts in CCs of the treated group. It seems that *γ‐oryzanol* protects CCs against oxidative stress and apoptosis. Cumulus cells surrounding oocytes have an important role in appropriate oocyte maturation. They produce main components essential for the development of oocytes in early embryos up to the EGA stage and also scavenge the inhibitory or toxic components around the oocytes (Russell et al. 2016). Upon our results, *γ‐oryzanol* affected the regulation of proapoptotic genes in CCs during the process of maturation to protect them against apoptosis and assist in accomplishing their supportive role.

## 5 Conclusion

*γ‐Oryzanol* improved the development of ovine oocytes to the blastocysts through an antioxidative pathway, in which *γ‐oryzanol* enhanced the potential membrane of mitochondria and GSH level, decreased ROS level, upregulated *NRF2, SOD, CAT, GPX* and *BCL‐2*, and downregulated *BAX and CASP‐9* transcription. This sustained the antioxidant cellular response, maintained a higher enzymatic activity, turned off the generation of ROS and provided a valid first line of defence against oxidative stress in ovine oocytes underwent maturation in vitro.

## Author Contributions

**Saeed Musapoor**: methodology, writing–review and editing. **Najmeh Davoodian**: conceptualization, supervision, methodology, investigation, writing–original draft, writing–review and editing, funding acquisition. **Ali Kadivar**: investigation, methodology, formal analysis, writing–review and editing. **Ebrahim Ahamdi**: investigation, methodology, writing–review and editing. **Hassan Nazari**: investigation, methodology, writing–review and editing.

## Disclosure

All the authors declare that they prepared the article in their personal capacity, and the present paper is not as an official representative or otherwise on behalf of a sanctioned government.

## Ethics Statement

No approval of research ethics committees was required since all the experiments were conducted with materials collected from slaughterhouse.

## Consent

All the authors contributed to the performance of the study. The final manuscript was read and approved by all the authors. All the authors gave their consent for the publication of the research.

## Conflicts of Interest

The authors declare no conflicts of interest.

## Data Availability

The datasets of the current study are available from the corresponding author on a reasonable request.
